# Major Role of Microbes in Carbon Fluxes during Austral Winter in the Southern Drake Passage

**DOI:** 10.1371/journal.pone.0006941

**Published:** 2009-09-14

**Authors:** Maura Manganelli, Francesca Malfatti, Ty J. Samo, B. Greg Mitchell, Haili Wang, Farooq Azam

**Affiliations:** 1 Istituto Superiore per la Prevenzione e la Sicurezza del Lavoro (ISPESL) - DIPIA, Monteporzio Catone (RM), Italy; 2 Marine Biology Research Division, Scripps Institution of Oceanography, University of California San Diego, La Jolla, California, United States of America; 3 Integrative Oceanography Division, Scripps Institution of Oceanography, University of California San Diego, La Jolla, California, United States of America; Universidad Miguel Hernandez, Spain

## Abstract

Carbon cycling in Southern Ocean is a major issue in climate change, hence the need to understand the role of biota in the regulation of carbon fixation and cycling. Southern Ocean is a heterogeneous system, characterized by a strong seasonality, due to long dark winter. Yet, currently little is known about biogeochemical dynamics during this season, particularly in the deeper part of the ocean. We studied bacterial communities and processes in summer and winter cruises in the southern Drake Passage. Here we show that in winter, when the primary production is greatly reduced, *Bacteria* and *Archaea* become the major producers of biogenic particles, at the expense of dissolved organic carbon drawdown. Heterotrophic production and chemoautotrophic CO_2_ fixation rates were substantial, also in deep water, and bacterial populations were controlled by protists and viruses. A dynamic food web is also consistent with the observed temporal and spatial variations in archaeal and bacterial communities that might exploit various niches. Thus, Southern Ocean microbial loop may substantially maintain a wintertime food web and system respiration at the expense of summer produced DOC as well as regenerate nutrients and iron. Our findings have important implications for Southern Ocean ecosystem functioning and carbon cycle and its manipulation by iron enrichment to achieve net sequestration of atmospheric CO_2_.

## Introduction

Southern Ocean plays a crucial role in the global carbon cycle, exerting a major control on atmospheric CO_2_ concentration [1], hence the need to understand the role of biota in the regulation of carbon fixation and cycling. Southern Ocean is a large and heterogeneous biogeochemical system. The coastal and ice edge waters experience intense spring and summer blooms–mainly diatoms and *Phaeocystis*–that support a food web via zooplankton and krill to penguins and whales. In contrast, the open ocean is the largest high-nutrients low-chlorophyll (HNLC) area, characterized by low primary productivity (PP), mainly iron-limited, as demonstrated by several iron enrichment experiments [2] and observations of blooms induced by natural iron fertilization [3]–[4]. Iron fertilization to stimulate net phytoplankton growth has been proposed as one method for mitigating rising atmospheric CO_2_ [5]. Yet, the fate of the carbon accumulating in the productive areas during summertime or in artificially fertilized areas is critical to how biological forces make the Southern Ocean a source or a sink for carbon. There is much interest in whether the southern ocean food web is dominated by microbial loop processes–as in tropical and temperate oligotrophic ocean [6]–[7]. Previous studies showed weak bacteria-PP coupling and ascribed this to temperature restriction of dissolved organic matter (DOM) utilization [8], limited DOM availability [9] or strong grazing pressure [9]–[10]. However, others found that bacteria process a significant fraction of primary production during bloom conditions by consuming the phytoplankton derived dissolved organic matter [11]–[12]. An important feature of the Southern Ocean is the strong seasonality, due to long dark winter with minimal PP. Few studies done in autumn-winter in marginal se-ice edge zone [13]–[15] and in the Drake Passage [16], found substantial bacterial activity; however they did not explicitly address microbial loop processes, and were limited to the surface mixing layer [13]–[15]. Direct evidence of a significant role of the microbial loop over a broad area and in the mesopelagic layer in winter is still lacking.

**Figure 1:**
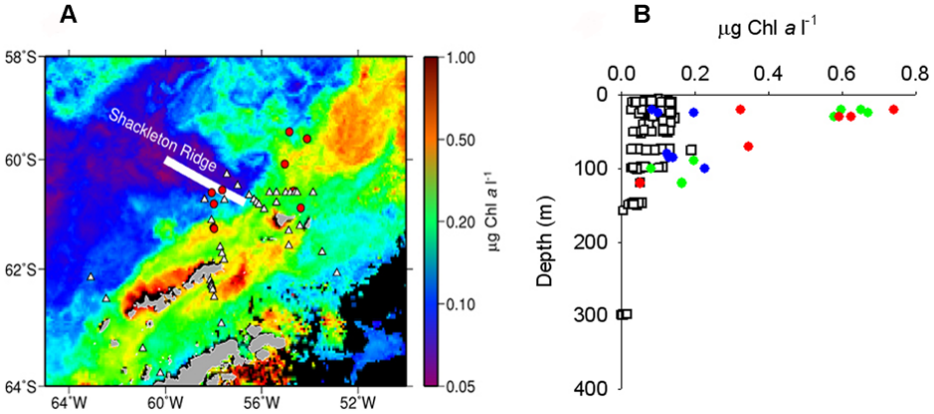
Sampling area and chlorophyll *a* distribution. (Panel A) MODIS-Aqua composite image of the chlorophyll-*a* gradient during 2004 summer. Red dots: summer 2004 sampling stations; white triangles: winter 2006 sampling stations. (Panel B) Depth profile of chlorophyll-*a* (*Chl a*) from summer 2004 cruise (blue circles = ACC water; green circles = mixed water; red circles = shelf water) and winter 2006 cruise (empty squares).

In summer (2004) and winter (2006) cruises in Southern Drake Passage (Fig. 1a) we studied microbial communities and processes during sharply contrasting productivity regimes. Our goal was to address the significance of the microbial loop in carbon fluxes and food web dynamics particularly in supporting the food web in winter.

## Results and Discussion

**Figure 2:**
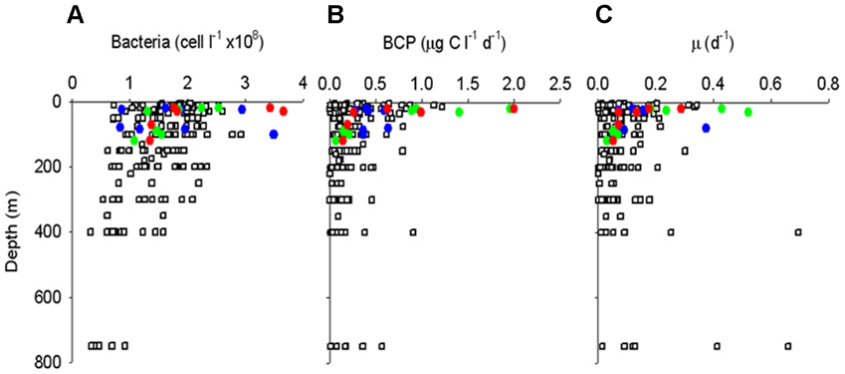
Depth profile of bacterial parameters in summer 2004 and winter 2006. Winter data are from 32 stations from 5 to 400 m; at 6 stations samples were also taken from 750 m. Summer: blue circles = ACC water; red circles = shelf water; green circles = mixed water; empty square = Winter. (Panel A) Bacterial abundance; (panel B) BCP = bacterial carbon production; BCP calculated from ^3^H-Leucine incorporation, employing a conversion factor of 3.1 kg C per mol of Leu [17]; (panel C) μ = bacterial growth rate. Cell-specific growth rate calculations assumed 20 fg C per cell [18].

In summer (February 12-March 24 2004) we sampled the epipelagic layer (∼67 m). Our study area had strong gradients of chlorophyll-*a* (*Chl a*) (0.05 to 0.74 µg *Chl a* L^−1^) from oligotrophic Antarctic Circumpolar Current (ACC) waters to east of Shackleton Transverse Ridge (STR) (Fig. 1a, b). The ACC current is steered south by STR and mixes with the iron rich Shelf water, stimulating high PP downstream [4]. Bacterial abundance (1.9×10^8^±0.9×10^8^ L^−1^ s.d.), bacterial carbon production (BCP) (0.63±0.57 µg C L^−1^d^−1^ s.d.) and growth rates (μ) (0.16±0.14 d^−1^ s.d.) (Fig. 2) were in the low end of the range for other oligotrophic regions [19]. They were positively correlated with *Chl a* (Spearman R, p<0.05, BCP vs *Chl a* R = 0.49, μ vs *Chl a* R = 0.46, n = 21). Carbon flux into bacteria as fraction of co-local PP is a measure of the strength of bacteria-organic matter (OM) coupling. In summer, BCP was equivalent to ∼30% of PP in low chlorophyll ACC waters and ∼13% in high PP mixed water. Assuming a conservative growth efficiency (BGE) of 36% [20] the bacterial carbon demand (BCD) over the whole area would be ∼40% of PP (Table 1). Thus, bacteria-OM coupling was robust and comparable to other oligotrophic and mesotrophic waters. (Yet, it would spare ∼1/2 of PP, some of which could accumulate and support the wintertime food web).

**Table 1:** Areal data from summer 2004 and winter 2006 cruises.

		Number of stations	Depth of integration (m)	PP (mg C m^−2^ d^−1^)	BCP (mg C m^−2^ d^−1^)	Summer excess DOC (g C m^−2^)	BCD* (mg C m^−2^ d^−1^)	BCD** (mg C m^−2^ d^−1^)
Summer	Epipelagic	10	65 (17)	354.8 (197.1)	51.9 (30.7)	37.4 (18.8)	143.1 (87.0)	199.5 (118.1)
	Mesopelagic	1	685§	–	N.D.	82.8§	N.D.	N.D.
Winter	Epipelagic	38	156 (75)	7.6 (5.4)	25.2 (21.3)	°	65.2 (55.0)	203.4 (171.7)
	Mesopelagic	38	600 (64)	–	84.5 (90.5)	°	227.2 (249.0)	681.2 (729.4)

All parameters for the summer cruise have been integrated over the entire euphotic layer (1% incident PAR at surface; it was similar to the mixed layer depth or deeper). The winter PP has been integrated over the euphotic layer while other parameters have been integrated over the mixed layer depth (epipelagic) and from there down to 750 m (mesopelagic layer). Averages and standard deviation in parenthesis.

PP = primary production; BCP = bacterial carbon production; Summer excess DOC = summer dissolved organic carbon values minus average winter constant value (36.5±2.8 µM C s.d.); BCD = bacterial carbon demand (BCP/bacterial growth efficiency); N.D. = not determined.

* BCD calculated using a bacterial growth efficiency derived by the curve in ref. [20] (∼36% in summer and 39% in winter).

** BCD calculated using bacterial growth efficiency of 13% in summer and 6.2% in winter (averaging all data for summer and only HNLC for winter from [12]).

§ no s.d. reported because the value is derived from a single depth profile.

° DOC data for winter are not reported, since they are considered as constant refractory DOC values, and have been used to determine summer excess DOC.

In winter (July 3-August 15 2006) we occupied 38 stations and sampled depths from surface to 300–750 m (Fig. 1a). In the epipelagic layer, as expected, *Chl a* and PP were much lower than in summer (Fig. 1b and Table 1) yet, bacterial abundance was similar to summer and these populations were active in terms of μ and BCP (Fig. 2). Although our summer sampling was limited, the winter and summer ranges for both μ and BCP were comparable. Indeed, the winter bacterial abundance, BCP and μ in our study area were comparable to those reported for low latitude oligotrophic systems such as the central North Pacific gyre and Sargasso Sea. Bacteria were also abundant and active in the mesopelagic layer, and could mediate significant carbon flux. The mesopelagic bacterial abundances were within a factor of 2 and mesopelagic BCP and μ were comparable to their ranges in the epipelagic layer (Fig. 2). Notably, the high BCP at depth was due to higher μ (0.70 d^−1^ at 400 m; Fig. 2). Thus, wintertime bacterial populations throughout the epipelagic and mesopelagic layers were abundant, growing and displayed substantial carbon demand. Further, since PP was greatly reduced in winter the epipelagic PP was ∼3 fold *lower* than BCP and an order of magnitude *lower* than BCD (estimating epipelagic BCD at BGE ∼39% [20]) (Table 1). PP could therefore have supported only ∼10% of BCD and majority of BCD must have been met by sources other than the contemporaneous PP.

**Figure 3:**
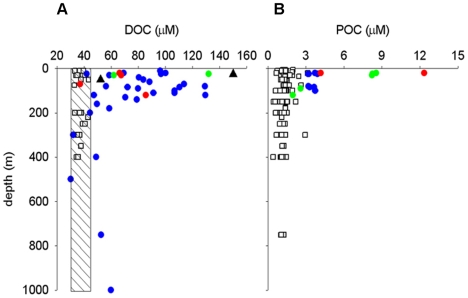
Depth distribution of organic matter pools during summer and winter cruises. Summer 2004: blue circles = ACC water; red circles = shelf water; green circles = mixed water; empty square = Winter 2006. (Panel A) DOC = Dissolved organic carbon. The data presented are measurements of total organic carbon, but since POC represents a negligible contribution to total organic carbon (POC represented only between 7% and 2% of the total pool) we can consider the analysis as DOC values. The shaded area covers the range of winter concentration. For a comparison, a range of variations of DOC from the FRUELA cruise study area in summer (Gerlache Strait, Bransfield Strait and Bellinghausen Strait) [22] has been reported (black triangles). (Panel B) POC = Particulate organic carbon.

We compared summer and winter data on areal basis to assess possible sources of carbon to support bacteria in winter (Table 1). SeaWiFS composite images of summer 2006 (Jan–Mar) for our study area showed a *Chl a* pattern similar to 2004; so we assume that summer 2004 values can be applied to summer 2006. Mesopelagic BCD would usually be met by sinking POC (or DOC mixing from surface [21]). In our area sinking particles from contemporaneous PP in winter could have supported only ∼3% of mesopelagic BCD even if, improbably, all PP sank into the mesopelagial. Since sinking POC could not have supported winter mesopelagic BCD, DOC is a likely source. DOC accumulated in the upper 200 m in the productive summer (Fig. 3a), presumably as slow-to-degrade components of phytoplankton production evade attack by summer bacterial communities. In winter DOC was very low (36.5±2.8 µM s.d.; Fig. 3a), in low range of constant refractory deep water DOC concentration [23]. The summer to winter DOC decline (37.4 g C m^−2^ in the epipelagic zone and 82.8 g C m^−2^ in the mesopelagic zone; Table 1) would suffice to support the observed BCD. In terms of carrying capacity the DOC drawdown could have supported the epipelagic BCD for ∼570 d and mesopelagic BCD for ∼365 d. Our calculations are based on two assumptions: the conversion factor (CF) from leucine incorporation to carbon production, and the growth efficiency. Regarding CF, we used a well constrained CF of 3.1 kgC mol leu^−1^ [17], that is the same value that [24] and [25] empirically determined in the Weddell-Scotia Sea and in the Atlantic sector of Southern Ocean. As for BGE, we used the model of [20], that relates temperature to BGE. The summer and winter values (36% and 39%) are close to 40% determined by [24] and in the range of values determined by [26] (9–38%) in the Ross Sea. However, since we did not determine experimentally the BGE for our system, we cannot exclude that using other values found in Southern Ocean, we could have had a different outcome. Applying a lower CF of 1.5 (assuming no dilution factor [12], this value is equivalent to the lower range of CF empirically determined by [27]) and a lower BGE of ∼13% in summer and ∼6% in winter, empirically determined in the Kerguelen Plateau, Southern Ocean [12], the resulting BCD is even higher (Table 1), and the excess summer DOC would have supported BCD for 184 days in the epipelagic layer and only 122 days in the mesopelagic layer. Integrating over the entire water column, the DOC drawdown would be 120.0 g C m^−2^, enough to support BCD for ∼136–410 d, depending on the BGE. The excess DOC drawdown may indicate a lower BGE since energy requirement to utilize semi-refractory DOM in winter may be higher. In the central Arctic ocean, in winter, [28] determined a growth efficiency of 0.5%, based on respiration incubation experiments. Alternatively, the excess DOC may have had other fate(s), like horizontal transport [22], vertical export [21], or such as POC → DOC transformation and currently unrecognized biotic sinks. If vertical mixing was the main mechanism of DOC removal from summer surface 200 m rather than biological mineralization [21], we would expect an increase in winter DOC concentration in the layer 200–750 m, which was not the case (integrated winter DOC value in the layer 200–750 m was 238.0 vs 265.5 g C m^−2^ in summer). Furthermore, DOC concentration in winter was homogenously very low (see above, Fig. 3a). However, since we sampled only in summer and winter, we cannot exclude the possibility of water masses sinking and being advected northward, earlier in autumn, thus exporting semi-refractory DOC [29].

Interestingly, the winter POC pool, although not sustainable by the meager surface PP, was persistent and vertically homogeneous (Fig. 3b). It may be comprised of refractory non-sinking organic matter. Alternatively, it may be produced dynamically from DOC by polymer cross-linking (above) and provide organic rich microenvironments for bacterial growth in winter. This would be consistent with significant bacterial growth rates and carbon cycling during winter.

In winter bacteria produced biomass at 110 mg C m^−2^ d^−1^ (Table 1), a source of protein rich biomass potentially available to all particle-feeding organisms (depending on the state of bacterial aggregation). Our data on protist and virus abundances from a limited number of stations representative of the whole area and all depths (not shown), support the hypothesis of a dynamic bacterial community channeling DOM-carbon to protists and on to larger animals [30]. A strong predation pressure is consistent with the observed low variability in bacterial biomass despite substantial growth. Protist abundance was 3.2×10^5^±0.9×10^5^ cell L^−1^ s.d. (in the range reported for summer in Southern Ocean [31] and in winter in ice-covered Arctic Ocean [32]). Assuming an ingestion rate of 1.3–5.5 bacteria HNF^−1^h^−1^ [32] protists would consume 18% to >200% of our winter BCP. Bacteria/protists ratio of ∼400 is within the range of other Antarctic surface waters where significant grazing rates have been measured [31], [33]. Viruses were also likely a significant source of bacterial mortality since they persisted at substantial populations (1.4×10^9^±0.4×10^9^ L^−1^ s.d.) comparable to a summer study in the Drake Passage [34]. Virus/bacteria ratio was 6–22, found typically in marine waters, including a summer study in Southern ocean that showed significant virus induced bacteria mortality [35]. Thus, BCP could have supported carbon flux to protists and possibly on to larger animals; and an active viral loop that might enhance system respiration [35]–[36].

**Figure 4:**
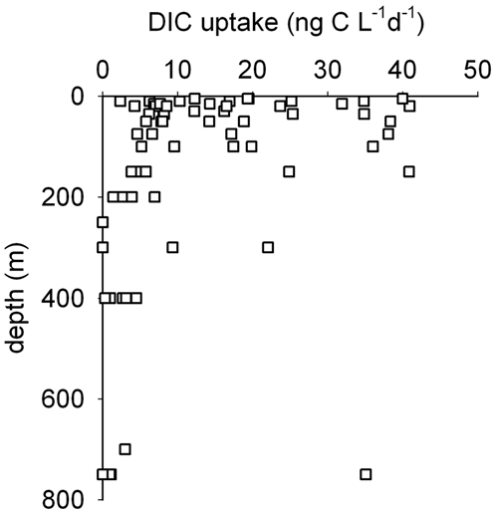
Depth profile of chemotrophic carbon production in winter 2006. DIC = dissolved inorganic carbon.

We considered that in addition to heterotrophic BCP there might also be chemoautotrophic production [37], probably due to *Archaea* [37]–[38]. Dark ^14^CO_2_ assimilation in winter 2006 showed significant but highly variable carbon fixation (Fig. 4), 0.1–40 ng C L^−1^d^−1^ corresponding to 8.8±15.8% s.d. of the total prokaryotic carbon production (bacterial and archeal). For the near surface experiments we cannot exclude a potential contribution of dark CO_2_ fixation by phytoplankton; however this is probably insignificant, since the incubations lasted from 55 to 100 h and further we did not find a significant correlation between DIC uptake and *Chl a* (not shown). While our measurements were limited it would be important to determine whether chemoautotrophic production in winter is a significant factor in Southern Ocean carbon cycle in a broader geographical context.

We hypothesized that sustained bacterial growth in winter at the expense of DOC semi-refractory to the summer population might have been accompanied by a shift in bacterial community composition as it has been observed in more productive areas [39]–[40]. Also, bacterial community structure can be influenced by top-down control; grazing and viral lysis can have a different impact on diverse taxa [41]. Whether the summer prokaryotic assemblages persisted into winter and whether there was a spatial variability was addressed by denaturing gradient gel electrophoresis (DGGE) and 16S rRNA sequence analysis of excised bands for both *Bacteria* and *Archaea*.

**Figure 5:**
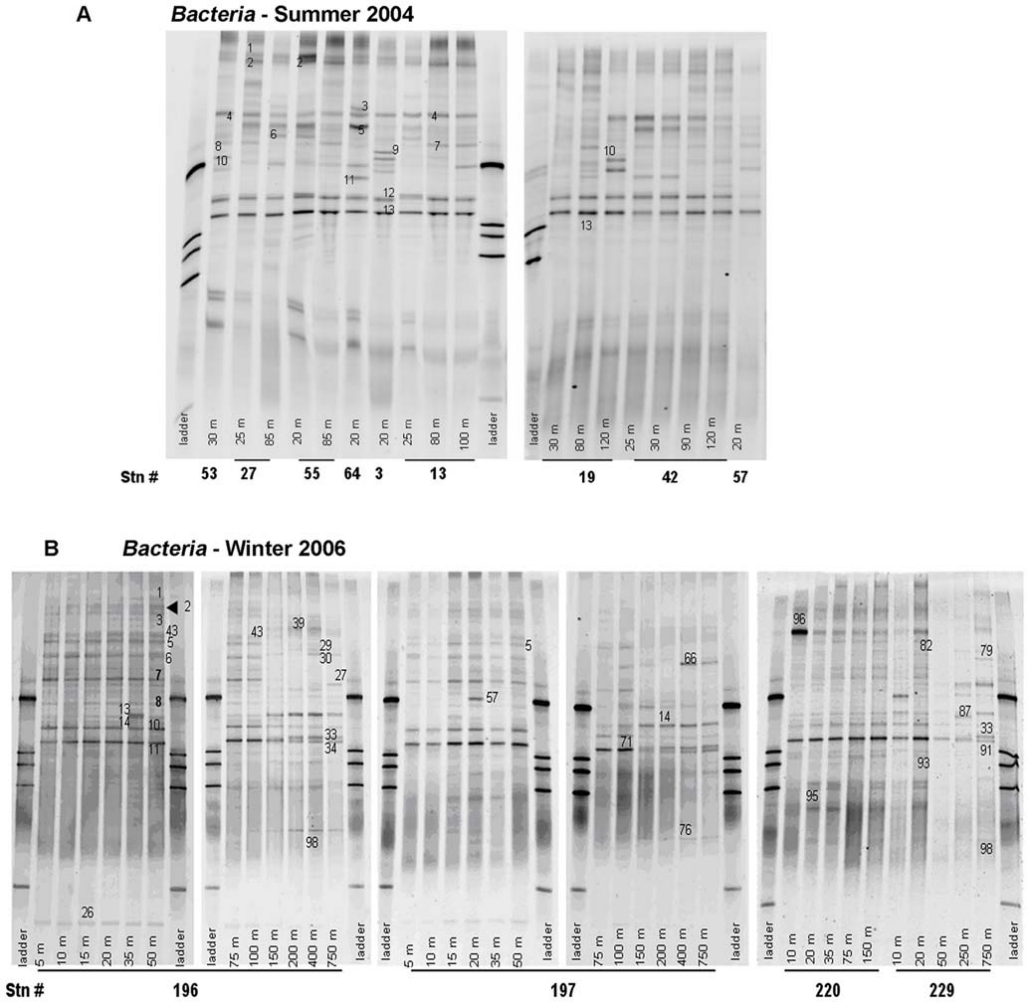
DGGE fingerprint of *Bacteria* amplicons from Summer 2004 (A) and Winter 2006 (B) samples. The numbered bands have been excised for sequencing.

*Bacteria* communities were different in summer and winter (Fig. 5, Table 2). In summer, in the epipelagic layer (9 stations, 20–120 m, Fig. 5A, Table 3), we detected only seven *Bacteria* phylotypes (and six *Eukarya* plastids)— three *α-Proteobacteria* (associated to *Roseobacter*), three *Cytophaga*-*Flavobacteria*-*Bacteroidetes* (CFB) and one *γ-Proteobacteria*. All had 99–100% sequence homology to known uncultured *Bacteria* (Table 2). Winter epipelagic samples (0–150 m, Fig. 5B, Table 3) had much greater community richness—20 phylotypes—possibly reflecting molecular complexity of available substrates. All winter phylotypes had 98–100% sequence similarity to uncultured *Bacteria* from environmental clone libraries, majority (16/20) of them from Antarctic or Arctic waters. The seasonal variation was great. Only one phylotype, a *γ-Proteobacteria* (EF648172, displaying 100% sequence similarity to an uncultured Arctic bacteria) was present both in summer and winter epipelagic samples. [39], analyzing seasonal DGGE profiles in surface coastal waters near Anvers Island, found a striking difference between winter and summer assemblages. Repeating the analysis 6 years later, on samples covering an annual cycle, [47] found a reproducible seasonal trend, with significant differences between winter and summer communities.

**Table 2:** List of GenBank accession number and closest relative to 16S rDNA bacterial sequences obtained from DGGE.

Accession #	Band number	% identity	Accession # closest relative	Closest relative	Phylogenetic group	Ref.
Summer 2004						
EF127653	1	100	AF355051	Uncultured Cytophagales bacterium Arctic97A-14	Bacteroidetes	[42]
EF127654*	2	100	DQ184451	Uncultured γ proteobacterium clone SBI04_7 16S	γ Proteobacteria	[43]
EF127656	3	97	AY664327	Uncultured phototrophic eukaryote, chloroplast	Eukarya	
EF127657	4	99	EF491388	Uncultured eukaryote clone S2-72, chloroplast	Eukarya	
EF127659	5	99	EF414204	Uncultured phototrophic eukaryote clone MPWIC_C08	Eukarya	
EF127660	6	99	AM920863	Uncultured bacterium clone LV_38	Bacteroidetes	
EF127661	7	99	EU005787	Uncultured marine bacterium clone KG_A3_120m83 16S	Bacteroidetes	[44]
EF127662	8	98	AM747382	Unc. Marine eukaryote	Eukarya	
EF127663	9	100	EF395741	Uncultured phototrophic eukaryote, chloroplast	Eukarya	
EF127664	10	100	AM920858	Uncultured bacterium	Eukarya	
EF127666	11	99	AY794086	Uncultured Roseobacter sp. clone F1C79	α Proteobacteria	[45]
EF127667	12	99	EU496898	Unc Rhodobacteraceae bacterium clone RCA-ANTXVI/3-16	α Proteobacteria	
EF127668	13	100	AM921555	Uncultured bacterium 16S rRNA gene, clone LV_135	α Proteobacteria	
Winter 2006						
EF648172*	1	100	EF127654	Uncultured proteobacterium isolate DPEU02	γ Proteobacteria	
EF648173	2	99	EU005814	Uncultured marine bacterium clone KG_A3_120m110	Bacteroidetes	[44]
EF648174	3	99	EU005908	Unc mar bacterium clone KG_C11_100m18 16S RNA gene	γ Proteobacteria	[44]
EF648175	**5**	100	U70715	Uncultured prasinophyte clone OM5	Plastid	[46]
EF648176	6	100	EU005720	Uncultured marine bacterium clone KG_A3_120m16	γ Proteobacteria	[44]
EF648177	7	100	DQ906747	Unc marine bacterium clone AntCL2E12 16S RNA gene	Bacteroidetes	[47]
EF648178	8	100	AY628656	Uncultured prasinophyte clone LS-E12 chloroplast	Plastid	[48]
EF648179	10	100	AM921438	Unc bacterium partial 16S rRNA gene, clone SG_116	α Proteobacteria	
EF648180	11	100	DQ184424	Uncultured gamma proteobacterium clone SBI04_1 16S	γ Proteobacteria	[43]
EF648181	13	100	EF667989	bacterium Antarctica-16	γ Proteobacteria	
EF648182	14	100–99	AF355039	Uncultured delta proteobacterium Arctic95C-5	δ Proteobacteria	[42]
EF648183	26	100	DQ295238	Uncultured marine bacterium Ant4E12	Actinobacteria	[49]
EF648184	27	100	AJ551107	Psychrobacter sp. wp30 partial 16S rRNA gene, isolate wp30	γ Proteobacteria	
EF648185	29	99	DQ668584	Uncultured bacterium clone Arctic6-G12	γ Proteobacteria	[50]
EF648186	30	99	AF354606	Uncultured gamma proteobacterium Arctic96BD-19	γ Proteobacteria	[42]
EF648187	**33**	100	AM921265	Unc bacterium partial 16S rRNA gene, clone PB_53	α Proteobacteria	
EF648188	34	100	AM921120	Unc bacterium partial 16S rRNA gene, clone EI_91	α Proteobacteria	
EF648189	39	100	AF469345	Uncultured alpha proteobacterium CTD44B	α Proteobacteria	[51]
EF648190	43	98–100	DQ925854	Uncultured marine bacterium clone ANT10A4	γ Proteobacteria	[47]
EF648191	57	100	AF419359	Uncultured beta proteobacterium MoDE-9	β Proteobacteria	[52]
EF648192	66	100	AM921509	Unc bacterium partial 16S rRNA gene, clone ST_68	γ Proteobacteria	
EF648193	71	100	EU005838	Uncultured marine bacterium clone KG_A3_120m134	γ Proteobacteria	[44]
EF648194	76	100	DQ513056	Uncultured bacterium clone CTD005-4B-02	α Proteobacteria	[53]
EF648195	79	99	AY704387	Uncultured bacterium clone CTD005-74B-02	Bacteroidetes	[53]
EF648196	82	100	DQ906724	Uncultured marine bacterium clone AntCL1D8	γ Proteobacteria	[47]
EF648197	87	100	AY664361	Uncultured Pseudoalteromonas sp. clone JL-BS-K75	γ Proteobacteria	
EF648198	91	100		Uncultured bacterium clone 4C230441	α Proteobacteria	[54]
EF648199	93	98	AY027805	Aequorivita lipolytica Y10-2T	Bacteroidetes	[55]
EF648200	95	100	DQ184430	Uncultured gamma proteobacterium clone SBI04_175	γ Proteobacteria	
EF648201	96	98	EU878158	Unc phototrophic euk isolate DGGE gel band DL27-11	Plastid	
EF648202	98	100	DQ513065	Uncultured clone CTD005-31B-02	Actinobacteria	[53]

* phylotypes found in both seasons. Ref. = References of the closest relative.

**Table 3:** List and parameters of stations sampled for bacterial community analysis.

	Station ID	Range of sampling depth (m)	Range of T (°C)*	Chl a (μg L^−1^)	Bacterial abundance (cell L^−1^×10^8^)
Summer	13	25–100	−0.1–3.1	0.12–0.20	0.8–1.9
	27	25–85	−0.7–2.6	0.10–0.13	0.9–1.2
	55	20–85	−0.2–3.0	0.08–0.14	1.6–2.0
	42	25–90	−0.2–3.1	0.20–0.67	1.5–1.9
	64	20	3.1	0.60	2.2
	3	20	1.9	0.74	3.4
	19	30–120	−0.1–1.6	0.05–0.63	1.4–1.8
	53	30	1.5	0.59	3.7
	57	20	0.7	0.32	1.8
Winter	178	5–750	−0.7–1.9	0.04–0.13	0.3–2.1
	196	5–750	−0.8–1.7	0.01–0.17	0.4–2.1
	197	5–750	−1–1.7	0.01–0.17	0.4–2.4
	220	10–150	−1.3–−1.8	0.06–0.07	1.7–2.3
	229	10–750	−1.8–1.7	0.04	0.5–1.2

* positive temperatures in summer at surface, in winter at depth.

In our samples, also the relative contribution of phylogenetic groups changed between seasons: *α-Proteobacteria* and CFB, the dominant groups in summer, decreased to 5 and 15% respectively in winter, whereas *γ-Proteobacteria* increased to 50%. In winter, we also found one representative of *β-*, *δ-Proteobacteria* and *Actinobacteria* (and three *Eukarya* plastids). *Roseobacter* [56] and the CFB group [57] are commonly associated to phytoplankton bloom; different *Roseobacter* clusters are well adapted to variable condition of DOM availability [44] and have been shown to account for ∼20–25% of total bacterial production in summer [43]–[44]. CFB is also an important group in Antarctic marine waters and sea-ice microbial community, associated to algal blooms or high primary productivity [58]. So the dominance of *α-Proteobacteria* and CFB in summer can be related to the availability of freshly/recently produced organic matter. A higher relative abundance of *γ-Proteobacteria* in winter, and a parallel decrease of *α-Proteobacteria*, has been observed in the Arctic Ocean [42]. We were successful in culturing one of the *γ-Proteobacteria* phylotypes in winter (band 13, EF648181) which is associated to *Alteromonadales*. Also this group has been found only in winter in Arctic waters [42]. Other studies in the Southern Ocean analyzed alternatively the summer [44] or the winter community [47], and found that *γ-Proteobacteria* and *α-Proteobacteria* were similarly abundant. The differences can be due to the fact that [44] analyzed bacterial community inside and outside a natural iron fertilized summer bloom, above the Kerguelen plateau, Southern Ocean, whereas we sampled in late summer, at the end of the productive season. [47] sampled from a coastal station, that can be very different from the pelagic environment. Furthermore, the *α-Proteobacteria* phylotypes were associated mostly to *Pelagibacter* and SAR11.

In winter we also analyzed mesopelagic samples (200–750 m, Fig. 5b, Table 3). Only 3 out of 14 *Bacteria* phylotypes were common with the winter epipelagic phylotypes; the bands unique to the mesopelagic communities displayed 99–100% sequence similarity with uncultured environmental bacteria from Arctic and Antarctic Ocean or from deep-sea samples, from seawater surrounding hydrothermal vents (Table 2). In mesopelagic waters *α* and *γ−Proteobacteria* phylotypes alone accounted for 79% of community diversity (43 and 36% respectively). Our findings are consistent with the few data available for deep ocean bacterial community structure, that found different phylotypes, related to depth [59]–[60].

**Figure 6:**
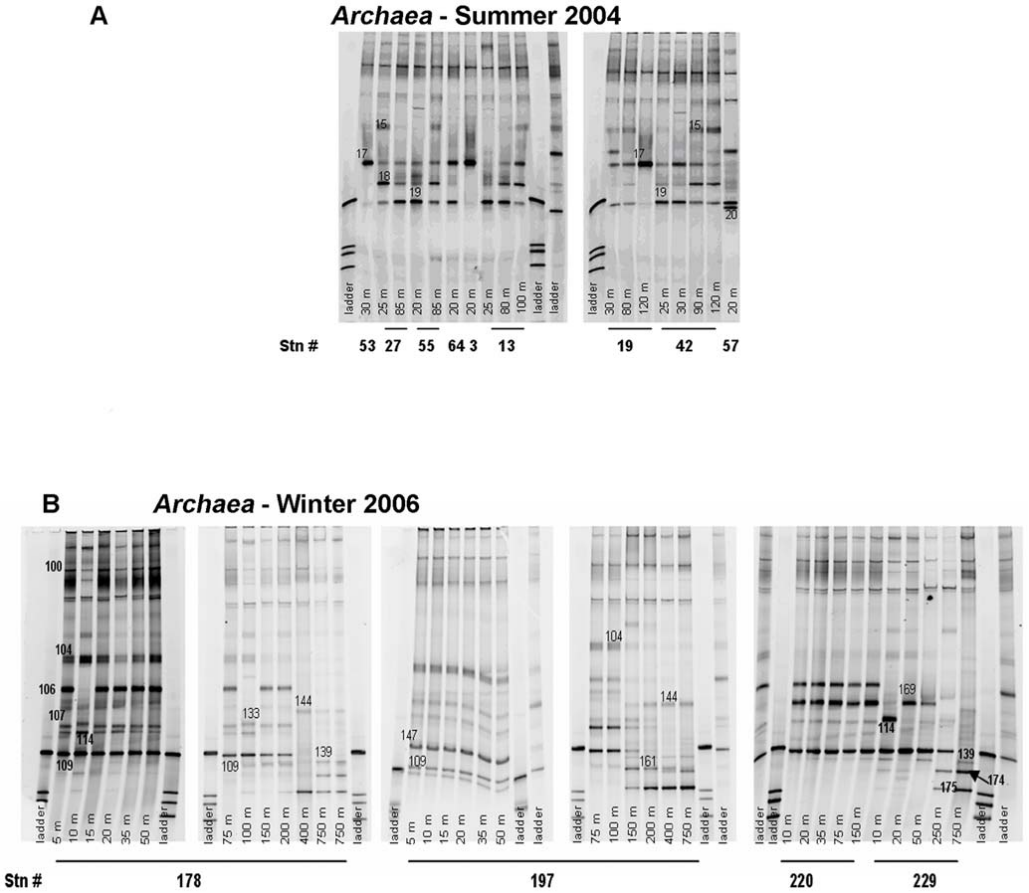
DGGE fingerprint of *Archaea* amplicons from Summer 2004 (A) and Winter 2006 (B) samples. The numbered bands have been excised for sequencing.

We might expect *Archaea*, which exploit different niches [39]–[40], to respond differently than *Bacteria*. In summer, in the epipelagic layer, we detected 6 archaeal phylotypes compared with 12 in winter (Fig. 6, Table 4). As was the case for *Bacteria,* archaeal community richness was also greater in winter than in summer. However, unlike *Bacteria,* all 6 summer archaea phylotypes were common to both seasons (Table 4). Five of 6 were *Euryarchaeota* and one was *Crenarchaeota*, and all 6 showed 97–98% sequence similarity to uncultured Arctic or Antarctic *Archaea*. Six *Archaea* unique to winter were all *Euryarchaeota* and showed 97–99% sequence similarity to uncultured Arctic and Antarctic *Archaea*. Of the 12 epipelagic *Archaea* in winter 9 were present also in the mesopelagic zones, where we also found 2 phylotypes unique to the mesopelagic zone (98% sequence similarity to uncultured Arctic *Archaea*).

**Table 4:** List of GenBank accession number and closest relative to 16S rDNA archaeal sequences obtained from DGGE.

Accession #	Band number	% identity	Accession # closest relative	Closest relative	Phylogenetic group	Ref.
Summer 2004						
EF640719*	15	98%	DQ146734	Uncultured archaeon clone iBSZ2f.80	Euryarchaeota II.a	[61]
EF640720*	16	97%	EU486954	Uncultured crenarchaeote clone FB04aw.90	Crenarchaeota	
EF640721*	17	98%	EF486592	Uncultured euryarchaeote clone M05a039.03	Euryarchaeota II.a	[62]
EF640722*	18	97%	EU199752	Uncultured euryarchaeote clone SCICEX122424H3	Euryarchaeota	
EF640723*	19	98%	EU199730	Uncultured euryarchaeote clone ANT9515E6	Euryarchaeota	
EF640724*	20	98%	AY288394	Uncultured euryarchaeote 97D-131-22A	Euryarchaeota II	[63]
Winter 2006						
EF640725	100	98%	EU199727	Uncultured euryarchaeote clone Ant9406E8	Euryarchaeota	
EF640726*	104	100%	EF640719	Uncultured marine archaeon clone DPAR02	Euryarchaeota	
EF640727*	106	100%	EF640720	Uncultured marine archaeon clone DPAR03	Crenarchaeota	
EF640728	107	98%	EU199759	Uncultured euryarchaeote clone SCICEX1231220E10	Euryarchaeota	
EF640729*	109	100%	EF640723	Uncultured marine archaeon clone DPAR06	Euryarchaeota	
EF640730*	114	100%	EF640722	Uncultured marine archaeon clone DPAR05	Euryarchaeota	
EF640731	133	99%	AY856360	Uncultured archaeon clone CTD005-2A	Euryarchaeota III	[64]
EF640732*	139	100%	EF640724	Uncultured marine archaeon clone DPAR07	Euryarchaeota	
EF640733	144	97%	AY288408	Uncultured euryarchaeote 97E-131-20	Euryarchaeota	[63]
EF640734	147	99%	AY288403	Uncultured euryarchaeote 97D-235-10	Euryarchaeota	[63]
EF640735	161	97%	AF257277	Uncultured marine euryarchaeote DH148-W1	Euryarchaeota II	[65]
EF640736*	169	100%	EF640721	Uncultured marine archaeon clone DPAR04	Euryarchaeota	
EF640737	174	98%	AY288381	Uncultured euryarchaeote 95B-131-15H	Euryarchaeota II	[63]
EF640738	175	98%	AY288407	Uncultured euryarchaeote 97E-131-15	Euryarchaeota II	[63]

* phylotypes found in both seasons. Ref. = References of the closest relative.

The abundance and diversity of Archaea in the Southern Ocean, has been widely documented [39]–[40], [66]. Our results are in agreement with the evidence that both Crenarchaeota and Euryarchaeota are less diverse than marine bacterial phylogenetic group [67]. Previous studies by FISH reported that Crenarchaeota were the dominant group of Archaea in waters off Antarctica Peninsula [40], but a successive study in the Antarctic Circumpolar Current [38], with the same technique and a better protocol of permeabilization of cell wall, showed a higher percentage of Euryarchaeota between 500 and 1000 m, of about 10–20% of total prokaryotes, about twice the previous estimate. Also [63], from a clone library from Arctic samples detected a number of Groups II (Euryarchaeota) clones similar to the Group I (Crenarchaeota). [63] from DGGE analysis never retrieved a sequence of Crenoarchaeota. The authors attributed this to a mismatch in the universal 517r primer, used for DGGE. We detected one Crenarchaeota, and the band is also very intense when present. It is possible that the nested PCR that we performed, increasing selectively the abundance of template DNA, reduce this problem. We are aware of several potential limitations of DGGE, such as chimeras and heteroduplex formation or PCR biases [68], but this technique was very useful for our purposes of relative comparison between different assemblages.

Both *Crenarchaeota* and *Euryarchaeota* have been shown to incorporate DIC [38] and this is consistent with our finding of DIC incorporation. The *Crenarchaeota* (EF640727) DGGE band was very intense in Shelf samples where DIC uptake was the highest. It would be interesting to determine if this *Crenarchaeota* is a significant autotrophic producer. Our results support the hypothesis that bacterial phylotypes are more affected by environmental variables associated with summer to winter transition than *Archaea* [40].

This study has shown that active and dynamic *Bacteria* and *Archaea* communities in Southern Ocean play a significant role in Antarctic food web and biogeochemical dynamics through the austral winter. They maintain substantial rates of secondary productivity during protracted periods of diminished primary productivity. They reset the system by organic matter decomposition and nutrient regeneration—setting the stage for the next summer. Our results have important implications for models of the functioning of Antarctic Ocean ecosystem and carbon cycle. The persistence of high microbial loop activity causing strong wintertime system net-heterotrophy is highly relevant to considerations of the efficacy of Southern Ocean iron enrichment to control the level of atmospheric CO_2_.

## Materials and Methods

Chlorophyll-*a* was determined by high performance liquid chromatography HPLC [69]. Net primary production was estimated by a modification of the Vertically Generalized Productivity Model (VGPM) [70]. The model was parameterized for the Antarctic temperature range considering a large dataset of ^14^C PP measurements, including ∼30 stations for each of our cruises.

Bacteria, viruses and protists were counted by epifluorescence microscopy. For bacteria and protists, 10 mL formaldehyde fixed samples were DAPI stained (3 µg/mL final concentration) and filtered onto a 0.2 µm polycarbonate black filter (Nuclepore). For viruses, samples were fixed with 0.5% paraformaldehyde, and stored at −80°C until analysis. 1 to 3 mL of samples were filtered onto 0.02 µm Anodisc filter and stained with SybrGreen [71].

Heterotrophic production was measured in dark incubations at *in situ* temperature, in trace metal clean labware, by ^3^H- leucine incorporation (20 nM concentration; 117Ci mmol^−1^) [72] in 4 hours incubations. The incorporated leucine was converted in carbon by a factor of 3.1 kgC mol Leu^−1^ [17]. Chemoautotrophic production was measured by DI^14^C incorporation in 50 mL duplicate samples, spiked with 10 to 50 µCi of sodium^14^C-carbonate (30–62mCi mmol^−1^) [38]. After 55 to 100 hours incubations, the samples were fixed with 4% final formaldehyde, filtered on 0.22 µm polycarbonate filters, rinsed twice with cold seawater and exposed overnight to a fume of concentrated HCl. Blanks (samples formaldehyde-fixed at T_0_) were subtracted from samples dpm.

Shifts in bacterial community composition were analyzed by polymerase chain reaction (PCR) amplification of a portion of the bacterial 16S rRNA gene, followed by DGGE and sequencing. Bacterial community DNA was extracted from 500 to 1000 mL of seawater filtered onto 0.2 µm pore size, 47 mm diameter, SUPOR-200 polyethersulfone filters (Pall). Filters were frozen at −80°C until extraction. DNA was extracted from the filters by the method of [73] with slight modifications. Briefly a quarter of a 47 mm diameter filter was placed in a Rnases and Dnases free tube and cells were lysed with freshly prepared lysozyme solution (1 mg mL^−1^, final concentration) in saline SET buffer (400 mM NaCl, 750 mM sucrose, 20 mM EDTA, 50 mM Tris-HCl, pH 9) for 30 min at 37°C. Proteinase K (100 µg mL^−1^, final concentration) and sodium dodecyl sulfate (SDS: 1%, final concentration) were added to the tubes and incubated for 12 h at 55°C. The supernatant was transferred in a fresh 15 mL PPCO tube (NUNC). The filter was washed with 1 mL of TE buffer (5 mM Tris-HCl, pH 7.5, 50 mM EDTA), which was then pooled with the lysate. 50 µg of t-RNA (Baker Roche Applied Science) per sample was added as a coprecipitant. To precipitate the DNA 1/10 of volume 3M NaAc (pH 5.2) and 2.5 volume 99.6% ethanol were added into the 15 mL tubes and incubated for 1 hr at −20°C. Samples were centrifuged at 20,000 x g, for 20 min, at 4°C to pellet the DNA. The pellet was washed with 70% ice-cold ethanol. Precipitated DNA was resuspended in MilliQ water and quantified fluorometrically (PicoGreen; Molecular Probes).

For Bacteria, a 16S rRNA gene fragment was amplified by PCR using a Eubacteria set, consisting of a universal primer complementary to position 517 to 534 (5′-ATTACCGCGGCTGCTGG-3′) and a bacterial primer complementary to position 341 to 358 with a 40-bp GC clamp (underlined) (5′-CGCCCGCCGCGCGCGGCGGGCGGGGCGGGGGCACGGGGGG CCTACGGGAGGCAGCAG-3′ [74]), following the protocol described in detail by [68]. After an initial denaturation for 5 min at 94°C, samples were amplified for 30 cycles by touchdown PCR [75]. For each cycle, denaturation was at 94°C for 1 min. Annealing was for 1 min at a temperature which decreased 1°C every two cycles from an initial 65°C to a final of 50°C. Primer extension was at 72°C for 3 min in each cycle, with a final 7-min extension following the last cycle. A negtive control, in which the template was replaced with an equal volume of sterile water, was included in each batch of PCRs.

For Archaea, 16S rRNA gene fragments ∼190 bp long for DGGE analysis were generated by nested PCR. The first PCR was performed using specific 21f (TTC CGG TTG ATC CYG CCG GA) and 958r (YCC GGC GTT GAM TCC AAT T) primers at the same PCR conditions described by the author: 30 cycles with a denaturation step at 95°C for 1.5 min, an annealing step at 55°C for 1.5 min, and the extension at 72°C for 1.5 min [76]. The resulting PCR products were used as templates for the second PCR, which was performed with an Archaea-specific 344f with a 40 bp GC clamp (underlined) (5′-CGCCCGCCGCGCCCCGCGCCCGTCCCGCCGCCCCCGCCCC ACGGGGCGCAGCAGGCGCGA-3′) and a universal 517r (5′-ATTACCGCGGCTGCTGG-3′) [63], at the same reaction conditions used for Bacteria.

Between two hundred - five hundred nanograms of PCR product were loaded on 8% polyacrylamide gels (acrylamide-N,N'-methylenebisacrylamide [37∶1]) containing denaturant gradients of 30 to 60% from top to bottom for Eubacteria and 20–55% for Archaea top to bottom (where 100% is defined as 7 M urea and 40% [vol/vol] formamide). Electrophoresis was performed with a hot-bath denaturing gradient gel electrophoresis (DGGE) unit (CBS Scientific, Del Mar, Calif.) using 0.5×TAE running buffer (20 mM Tris, 10 mM acetate, 0.5 mM Na2-EDTA, pH 8.2) at 60°C for 6 h at 200 V. Gels were stained for 30 min in SYBR Gold nucleic acid stain (1000× final concentration, Molecular Probes), destained for 10 min in 0.5×TAE, and photographed with UV transillumination. DGGE bands were excised using a sterile razor blade, and DNA was eluted overnight at 37°C in 200 µl of 1x SSC buffer (0.6 M NaCl, 60 mM trisodium citrate, pH 7). The eluant was centrifuged briefly to pellet acrylamide fragments. The supernatant was precipitated with 1/10 volume of LiCl and 3 volumes of ethanol, resuspended in 20 µl of Milli Q water and cloned using the Original TA cloning kit or the TOPO TA cloning kit (Invitrogen), following the instructions of the vendor. Insert-containing clones were identified by agarose gel electrophoresis after plasmid EcoRI digestion. DGGE profiles of reamplified, cloned DNA were used to check for heteroduplexes [77] and to confirm the positions of cloned bands relative to the original sample as described previously [68]. Unidirectional sequencing was performed by Agencourt, using M13 primers. Sequences were aligned to known sequences using BLAST (basic local alignment search tool) [78] All sequences were analyzed by the program CHECK_CHIMERA from the Ribosomal Database Project (RDP) [79]. The sequences submitted to GenBank are available under accession numbers listed in Table 2 and Table 4.

POC was measured with a CHN elemental analyzer, from samples collected by filtering 1–2 liters of seawater onto pre-combusted GF/F filters and stored in liquid nitrogen until analysis [4]. TOC samples were acidified (pH 2) with ultrapure HCl and stored in precombusted glass vials at 4°C until analysis with a Shimadzu TOC 5000A.

Statistical differences were tested with non parametric tests, with the software Statistica 6.0 (Statsoft); Kolmogorov-Smirnov test was applied to test differences between means and Spearman R to correlate two variables.
